# Application of a Force Sensor to Improve the Reliability of Measurement with Articulated Arm Coordinate Measuring Machines

**DOI:** 10.3390/s130810430

**Published:** 2013-08-13

**Authors:** Daniel González-Madruga, González Eduardo Cuesta, García Joaquín Barreiro, Ana Isabel Fernandez-Abia

**Affiliations:** 1 Department of Mechanical, Informatics and Aeroespatial Engineering, University of León, Campus de Vegazana, León 24071, Spain; E-Mails: danimadru@gmail.com (D.G.-M.); jbarg@unileon.es (J.B.G.); aifera@unileon.es (A.I.F.-A.); 2 Department of Manufacturing Engineering, University of Oviedo, Campus de Gijón, Asturias 33203, Spain

**Keywords:** AACMM, contact force sensor, probe deflection

## Abstract

A study of the operator contact force influence on the performance of Articulated Arm Coordinate Measuring Machines (AACMMs) is presented in this paper. After developing a sensor capable of measuring the contact force applied by an operator, a ring gauge has been used to analyse the relationship between the contact force and diameter and form errors measured with the AACMM. As a result, contact force has been proved as one of the main factors influencing the AACMM performance. A probe deflection model based on the Finite Element Method (FEM) has been also proposed in order to obtain the AACMM probe deflection caused by contact force. This allows measurement correction by comparing them with reference values, specifically, a ring gauge. Experimental test results show a significant measurement improvement that minimizes diameter error. Finally, an uncertainty evaluation for the contact force sensor and AACMM measurements with and without probe deflection model has been carried out in order to validate the ability of the sensor and the methodology followed.

## 1. Introduction

With the development of AACMMs, inspection tasks have evolved into a more efficient and flexible operation in manufacturing processes. Portability and the manual control nature of AACMMs allow fast setup and *in situ* measurements, giving them a relevant place in current industrial metrology. Furthermore, during last decade a few standards have settled AACMM evaluation tests [1,2]. In contrast with their strong potential, few works deal with AACMM issues and reliability.

AACMMs constitute their own kind of measuring machines with the combined features of both robot arms and CMMs. On the one hand, the kinematic structure of robot arms and AACMMs are similar, although AACMMs have only cylindrical joints with rotary encoders. Consequently, the authors have assumed the Denavit-Hartenberg (D-H) kinematic model, inherited from robot arm studies. AACMM calibration with the D-H model is not excessively complex and correction models can be added subsequently [3,4]. In contrast, robot arm features, structure designs and even their purpose (accuracy level, stiffness, control) are quite different from those of AACMMs, so AACMMs have to develop their own methodologies. Besides, CMMs' kinematic structure is very different from those of the AACMMs, although both of them collect point coordinates from a surface for inspection purposes. In addition, CMM performance has been widely studied and, therefore, a high level of reliability has been achieved, whereas AACMM performance has been poorly studied.

Another unique feature of AACMMs is their manual control, that adds measuring flexibility but also a non-predictable error source, the operator. Though the knowledge and experience of robot arms and CMM fields are used as a valuable support for AACMM studies, AACMMs require their own methodologies which must be related to the operator factor and the particular AACMM features.

Lastly, AACMM surveys focus on the calibration and verification of AACMMs. Santolaria *et al.* [3–7] proposed a calibration process based on the D-H kinematic model. Parameters of the kinematic model are calculated by measuring a ball bar gauge in several positions throughout the work volume. Such parameters and the rotation of the joints determine the coordinates of the contact points. This calibration minimizes the error of repeatability of the balls' centers and the distance error between ball centers by the least squares method. In a further step, these authors developed a correction model for repeatability [6]. Similar calibration methodologies have been proposed changing gauge, optimization method or gauge positions [7–16]. In some works authors develop special probes for the calibration task [3,7,10,17]. Such probes fit to the ball surface so many points are collected in a short time but, in this way, the actual probes (those used for actual part measurements) are deliberately excluded from calibration methodology.

Current AACMM calibrations allow us to know the overall error that affects its performance. However, the procedure is performed in a controlled environment, as a part of a procedure aimed to minimize the influence quantities. Calibration reduces the effects of the overall error which includes all error sources that affect AACMM performance. Few error sources have been studied although Vrhovec *et al.* [18] listed the main ones and quantified their approximate influence in AACMM accuracy. For example, AACMMs are expected to work in different temperature environments as a result of their portability. Because of that, Santolaria *et al.* [6] repeated the AACMM calibration at several temperatures and calculated the suitable kinematic model parameters that minimized the error at any temperature. Lin *et al.* [19] simulated the influence of encoders' systematic errors and how they propagate to the end point. Vrhovec *et al.* [18] also pointed out structure deflection as one of the main error sources since it depends on the AACMM design and a non-predictable factor, the operator. They proposed a laser-based deflection sensor that theoretically corrects this factor, but it was not implemented and calibration should be adapted in order to consider deflection correction. In a previous work [20], it was proved that AACMM performance is influenced not only by deflection and operator, but also by part surface and probe type.

Additionally, AACMM manual control results on a non-uniform and non-fully-controlled measurement process, as opposed to CMMs. The operator handles the AACMM and he has control, to some extent, of the measuring parameters: contact force and stability, point distribution, measuring strategy and probe orientation. This manual control causes worse values of repeatability and reproducibility. In fact, each contact point is commonly measured with a different set of measuring parameters even within the same geometric characteristic measurement. Furthermore, a point can be reached from almost infinite AACMM joints orientations which makes difficult to study the AACMM errors for each point. This way, operator factor becomes an important factor that affects AACMM performance through several measuring parameters. Some authors and standards cover this factor partially when they suggest AACMM joint orientations or analyse the rotation range for each encoder during calibration [1–3].

Measuring force affects the entire AACMM structure by deflecting the links and, therefore, separating the real geometry of the AACMM from its kinematic model. Besides the own weight of the AACMM structure, operator uses the measuring force for AACMM orientation control and contact with the part surface. Contact force is applied on the wrist joint and probe. As aforementioned, probes are usually excluded from calibration processes and, as a result, probe deflection is directly transferred to the measurement error. Even when actual probes are included in AACMM calibration, deflection caused by contact force is not corrected in the measurements. Cheng *et al.* [21] approached this problem with a simple device that calculates the probe parameter, but it does not compensate for probe deflection or errors during measurements.

Probes have been studied extensively in the CMM field. They are considered an important component of CMMs in terms of precision [22]. Many factors affect probe performance: probe geometry, contact detection system or measurement parameters as probe orientation or contact force [23–27]. Touch trigger probes, the most common type of probe used in CMM measurements, have been studied by theoretical models or experimental methods. Estler *et al.* [28] designed a model that includes the stylus bending besides probe geometry and kinematic of the contact detection system. Wozniak *et al.* [23–26] calculate the probe error using a high resolution transducer and a rotary table that simulated the CMM measurement. Nafi *et al.* [29] proposed a procedure to obtain probe errors by measuring a calibration sphere. Pereira *et al.* [30] improved the dynamic error of scanning probes by modelling the structure of the probe and measuring its error with a transducer on the part. From another point of view, Liang *et al.* [31,32] used strain gauges implemented on the probe and FEM to obtain the probe error. Even though the existing difficulty to control measuring parameters during AACMM measurements, some of these methods could be adapted to study the effects of operator, contact force and probe factors on AACMM performance.

This paper focuses on probe deflection as a result of contact force and the influence of operator factors by the development of a contact force sensor specifically designed for AACMMs [33]. A master ring gauge has been used to evaluate the relationship between operator and contact force factors with measurement error. Subsequently, a probe deflection model is proposed in order to reduce measurement errors.

## 2. Contact Force Sensor

**Figure 1.**
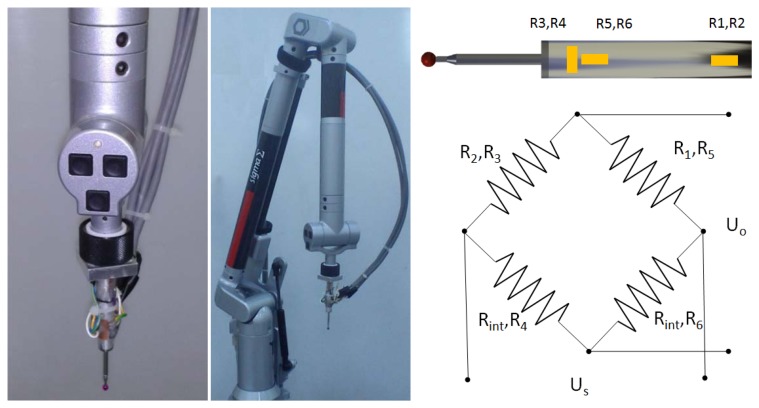
(**a**) Contact force sensor on the probe; (**b**) AACMM with the contact force sensor; (**c**) Strain gauges arrangement and circuit.

Strain gauges have been chosen as a suitable way to measure the contact force since they allow us to carry out a typical measurement with the AACMM. A contact force sensor has been implemented on an 85 mm long hard probe with a 4 mm ball (Figure 1). Although shorter probes are available, the higher surface of this probe enables mounting of strain gauges and a proper accessibility for ring gauge measurement.

### 2.1. Design and Implementation of the Contact Force Sensor

Contact force direction depends in part on surface and probe orientation [23–27], so it changes for each contact point. However, such a contact force has three components that can be measured by independent strain gauges circuits: one axial force component that causes shortening of the probe length and two bending force components that cause probe deflection. Because of the limited space, two strain gauges circuits were mounted on the probe surface: one for the axial force component and the other for one of the bending force components. Figure 1 shows the strain gauges circuit and their arrangement on the probe. Such a strain gauge arrangement is repeated on the other probe side in order to complete the circuit. Strain gauges R3, R4, R5 and R6 (1-XY13-3/350, HBM) are used to measure axial force with a full Wheatstone-bridge circuit and strain gauges R1 and R2 (1-LY13-3/350, HBM) are used to measure bending force with a half bridge circuit completed by the acquisition instrument (Rint).

Strain gauge circuits signals are collected by a data acquisition instrument (Dewetron 3021) at 1,000 Hz and their values are stored in a Matlab file for subsequent processing. This way, two sets of data are obtained from the measurements: contact force data and metrological data from the sensor and AACMM software (PCDMIS), respectively. The entire contact force sensor system was calibrated with standard weights that simulate the contact force of the AACMM. The maximum contact force calibrated was 13N.

### 2.2. Contact Force Parameters

**Figure 2.**
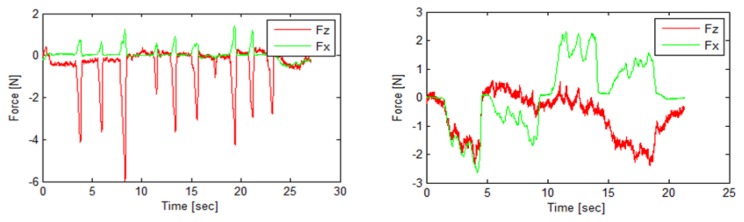
Examples of contact force acquired by the sensor.

The contact force sensor obtains real time data, Figure 2, which allows the study of the measurement process with AACMM and emphasises its difference with CMM contact force, Figure 2.

For probe deflection study, only the force in the instant of contact with a part surface is necessary, so data are processed as follows: firstly, data are considered in absolute values because direction depends on part surface and it is not necessary to study the level of contact force. Secondly, the instant when the AACMM reads the coordinates of contact points is determined using force maximum peaks. According to operator behaviour and experience, the contact force increases greatly at the moment of contact point reading and then it decreases. It can be explained as operator conduct to assure contact between probe and part. In the next step, the rest of contact force values are removed as well as the time scale, which is substituted by each point index. As a result, as many contact force values are obtained as number of contact points included in the point distribution. These values can be represented by significant values, for example the mean force, for using in the correlation with AACMM errors.

### 2.3. Uncertainty of Contact Force Measurement Sensor

Uncertainty of the system has been calculated following GUM [34] and EA-4/02 [35] recommendations. Combined standard uncertainty (*u_sensor_*) of the sensor is calculated from the contribution of those terms that add uncertainty to contact force measurement, Equation (1). These terms are standard uncertainties associated to the standard weight (u_stw_) used to calibrate the sensor, weights creep (u_cree_), resolution of the system (u_res_), hysteresis (u_hys_), sensor without load (u_Eo_), repeatability of the measurement of weights (u_rep_) and environment temperature (u_tem_) during the sensor calibration procedure, Equation (2):

(1) $$u_{\text{sensor}}^{2}=u_{\text{stw}}^{2}+u_{\text{cre}}^{2}+u_{\text{rep}}^{2}+u_{\text{hys}}^{2}+u_{Eo}^{2}+u_{\text{res}}^{2}+u_{\text{temp}}^{2}$$

(2) $$u_{\text{sensor}}=\sqrt{{\left(\frac{U_{\text{stw}}}{k}\right)}^{2}+\frac{M1_{e}^{2}}{12}+\frac{s{\left(x\right)}^{s}}{N}+\frac{H_{e}^{2}}{12}+\frac{E_{oe}^{2}}{12}+\frac{R^{2}}{12}+{\left(L\Delta Tu_{\text{CTE}}(\alpha )\right)}^{2}}$$

Uncertainty of the contact force sensor was determined by loading and unloading of well-known standard weights that simulate contact forces. Standard weights used for calibration and uncertainty evaluation are M1 class according to the OIML R111-1 [36] document. Some of the uncertainty terms are obtained by statistical calculation of repeated measurements (Type A), some others are calculated from calibration certificates, system or environment parameters and their probability distribution (Type B). The expanded uncertainty of a standard weight is 0.8 mg that is provided by its calibration certificate (coverage factor *k* = 2). Standard uncertainty of the creep of the standard weight uses the maximum permissible error (*M1_e_*) for M1 class weight [36] with a rectangular distribution. Repeatability uncertainty is determined by simulating the contact force with standard weights and checking the force reported by the sensor. Standard weights go from 10% of total calibrated weight (1.3 kg) to 100% with steps of 10% of total weight. Ten repetitions were performed for evaluating this uncertainty term. For each weight, the standard deviation of the mean values (s(x)/√n) was calculated by using deviations of the force readings. Then, the worst case was considered as the uncertainty of repeatability. For hysteresis uncertainty three weights were used: no weight, 50% and 100% of maximum calibrated weight. Furthermore, they were loaded and unloaded and the difference of measurement of the 50% weight was considered the hysteresis maximum error (*H_e_*) with a rectangular distribution. Sensor uncertainty without load is determined similarly to the hysteresis term. Difference of the measurement with no weight (*E_oe_*) and a rectangular distribution was used to calculate the standard uncertainty. Resolution uncertainty uses the resolution of the system (*R* = 0.001 N) and a rectangular distribution. Finally, temperature uncertainty is determined taking into account the maximum variation of the contact force measurement. This uncertainty considers the uncertainty of the coefficient of thermal expansion of probe material (*u_CTE_* = 1 × 10^−6^ °C) and the variation of measuring temperature from 20 °C during calibration (ΔT = 0.2 °C). The contribution of this factor is considered low, because the length of the gauges is short, gauges have a good response to temperature and contact force circuits exclude thermal effects. Finally, once the combined uncertainty is obtained, effective degrees of freedom are calculated according to the GUM document [34], given a coverage probability of 95%. Uncertainty evaluation was carried out for both axial and bending force (Table 1).

From the uncertainty evaluation, the expanded uncertainty of both force components is 0.0974 and 0.1518 N, which represents the worst case. These values are far enough from the maximum calibrated load, 13 N, and always above top operator force (which in common conditions never exceeds the value of 8 N).

## 3. Probe Deflection Model

**Figure 3.**
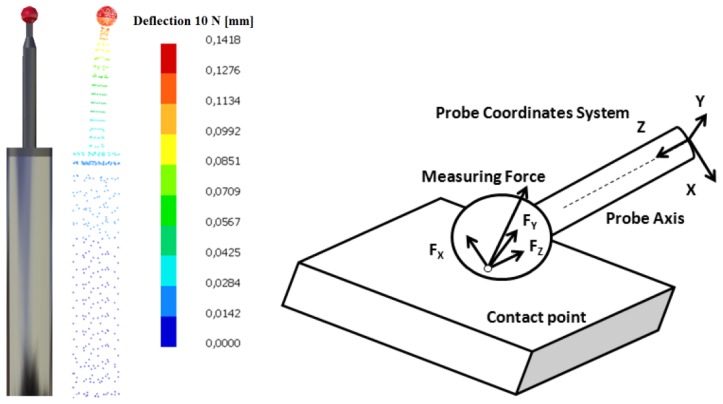
Probe deflection for 10 N contact force and contact forces scheme.

A contact probe causes deflection on the probe that modifies the coordinates of measured points. Some authors have studied the relationship between contact force and probe deflection with strain gauges and FEM [30,31]. The simple geometry of AACMM probes makes easy to study their response to the contact force by FEM. Thereby, probe geometry was modelled according to manufacturer technical specifications. In a further step axial force (F_z_) and bending force (F_x_) were applied on the stylus ball (Figure 3). For a 1 N axial force a shortening of 0.0000876 mm was calculated. The model was simulated with several forces (1, 5 and 10 N) in order to check linearity of the model. For a 1 N bending force a deflection of 0.0142 mm was calculated. Shortening is approximately 160 lower than deflection, which can be explained by probe geometry and contact force direction. Whereas common bending forces of 5 N cause a significant deflection on the probe and, therefore, a relevant measurement error, axial forces of 5 N barely affect to AACMM measurements. This way, although axial forces can be useful for AACMM performance evaluation, their effects can be neglected.

**Figure 4.**
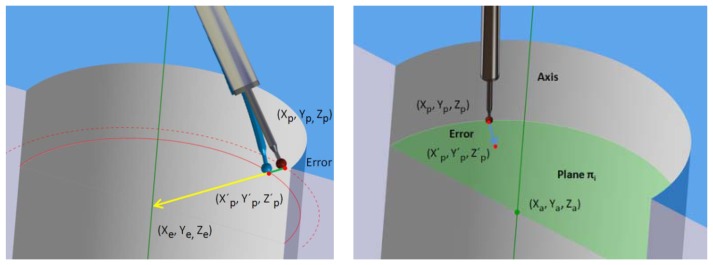
(**a**) Probe deflection explanation; (**b**) Error direction for correction model.

Once deflection is known, a correction model that subtracts such an error from the contact point coordinates is possible. However, direction of deflection is unknown due to the incompleteness of the contact force sensor [two of the three contact force components are measured; for this reason, the ring gauge geometry and the way to measure inner cylinders are taken into account in the design of the probe deflection model, Figure 4a]. Inner cylinders require a parallel strategy of measurement between cylinder and probe axis, so the main error is caused by probe deflection. Furthermore, the deflection error occurs towards the cylinder axis. This error direction is assumed by the model. Since the probe is deformed, theoretical coordinates of the centre of stylus ball are located on a larger diameter of the ring gauge. This also explains the larger diameter found in test results.

From ring gauge measurement, coordinates of contact points and ring gauge geometry data are obtained (Figure 4b). The AACMM measurement software (PCDMIS) uses the coordinates of contact points to construct the cylinder geometry associated to them by the least squares method. Since contact point coordinates will be modified, this construction method is reproduced in Matlab in order to compare cylinder geometry after using the probe deflection model. This geometry construction method was tested with initial coordinates values and results agreed with measurement software.

The error direction is a vector that goes from each contact point to the cylinder axis and it is perpendicular to this one. First, the plane π_i_ for each point is determined by Equation (3):

(3) $$\pi_{i}:\;A_{i}\;x+B_{i}\;y+C_{i}\;z+D_{i}=0$$

where A_i_, B_i_ and C_i_ are the axis vector components (i, j, k) of the cylinder because it is perpendicular to the plane *π*_i_. As each point coordinates (X_p_, Y_p_, Z_p_) belong to this plane, D coefficient can be calculated as Equation (4):

(4) $$D_{i}=-iX_{P}-jY_{P}-kZ_{P}$$

Next, the intersection point of the cylinder axis (X_a_, Y_a_, Z_a_) and the plane is calculated, Equation (5):

(5) $$(X_{a},Y_{a},Z_{a})=\pi_{i}\cap \text{Cylinder axis}$$

Then, the error direction vector is determined by the intersection point and the contact point coordinates, Equation (6). Then, it is normalized, Equation (7):

(6) $$\text{Error direction vector}=(X_{a}-X_{P},Y_{a}-Y_{P},Z_{a}-Z_{P})$$

(7) $$\text{Normalized error direction vector}=(i_{E},j_{E},k_{E})$$

Finally, the probe deflection values are added to the error direction to complete the probe deflection model, Equation (8):

(8) $$\left(X\prime_{P},Y\prime_{P},Z\prime_{P})=(X_{P},Y_{P},Z_{P})+\left|d\right|\cdot (i_{E},j_{E},k_{E}\right)$$

Deflection (*d*) is obtained from the previous FEM probe analysis and the contact force sensor (*f*). Since, only bending force is used in the model it is calculated as indicated in Equation (9):

(9) d=f⋅0,0142

Several probe deflection models can be implemented according to the force parameter used. One of the tested models uses the contact force measured for each point, that is, each contact point coordinates is corrected with the force used to take them. Other tested models use the same contact force value to correct all of the contact point coordinates. The considered force values were the *mean*, the *maximum* and the mean of the 10 maximum point forces (*10Max*) of the contact point forces. These force parameters were chosen as relevant force values. Next section discusses the main results obtained with the different models.

## 4. Tests and Results

**Figure 5.**
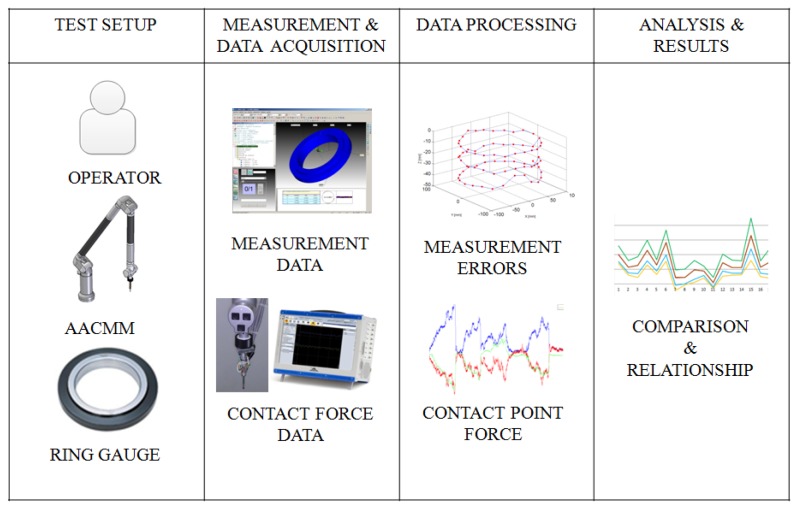
Test methodology.

In order to analyze the influence of operator and contact force on the AACMM performance, a ring gauge was measured with the contact force sensor already described. Figure 5 shows the followed methodology. This test methodology allows analysing the influence of contact force on measurement errors when using AACMMs.

Test setup is defined by an operator who measures a ring gauge with an AACMM. Only one operator is required in order to avoid the influence of several operators on the AACMM performance. The master ring gauge is a Mitutoyo ring gauge with a diameter of 174.996 mm and a form error of 0.005 mm. The AACMM used is a 2018 Sigma Romer measuring arm with a range of 1.8 m and a hard probe, 85 mm long a 4 mm diameter ball.

Ring gauge was measured several times (17 accepted measurements) with a specific point distribution. In particular, 72 points were taken for measurement. These points were divided into three rows and spread uniformly, so points were spaced about 15°. Point distribution was maintained uniform, to a certain extent, by means of a grid that marked the suitable area (approximately 2 cm^2^) for contact points without interfering with the AACMM measurement.

Despite this grid, repeatability of contact points still cannot be compared to CMM repeatability. Because of that, reference values of ring gauge were taken by a CMM, simulating the AACMM repeatability within such area. Thus, several measurements were taken with the CMM varying successively each point location to cover the limits of the suitable area. Mean values of the repetitions were used as reference values for test. Additionally, contact force values for these reference values were considered 0 N. This methodology was developed in order to avoid other error sources than probe deflection. In this context, point coordinates data and contact force data were collected from each measurement on the ring gauge. After their processing the analysis was carried out.

### 4.1. Contact Force Analysis Results

**Figure 6.**
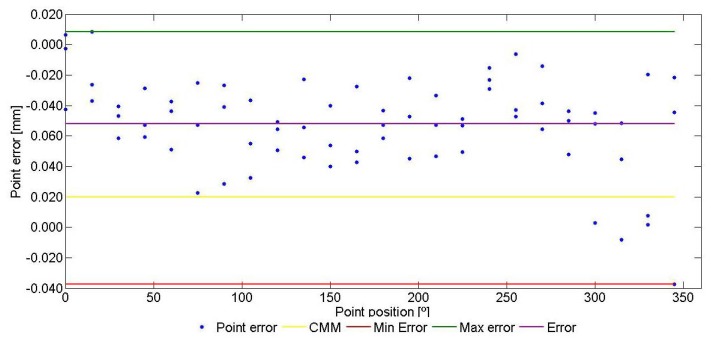
Example for contact point errors.

The contact point error is the difference between the distance of the contact point to the cylinder axis (radius value for that point) and the reference radius value (surface of the cylinder). An example for contact point errors of a cylinder measurement is showed, Figure 6. X axis represents the angular position of the point (0°–360°) within ring measurement.

Value 0 is the radius of the ring gauge measured with a CMM. The errors of the 72 contact points are displayed by their angular position. The radius error of the AACMM measurement is calculated as the mean value of contact point errors and it is equal to the radius value given by the measurement software. Obviously, diameter error for the ring gauge is double the radius error. The maximum and minimum radius can be calculated using the maximum and minimum contact point errors. Ring gauge form error measured with AACMM is the difference between both values. As can be observed, the majority of contact point errors are positive, which means a larger diameter in AACMM measurements.

**Figure 7.**
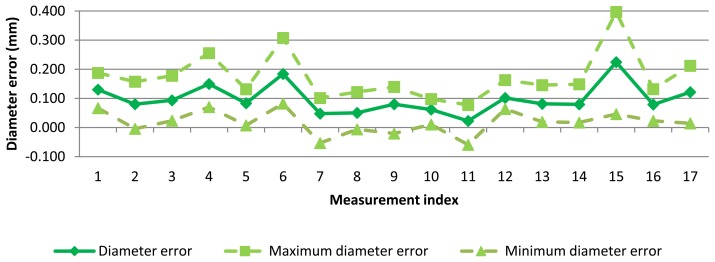
Diameter error for test measurements with AACMM.

Diameter error for each measurement can be seen in Figure 7. As expected, variability of AACMM measurements is considerable. A mean diameter of 0.100 mm has been found but diameter error reached a maximum peak higher than 0.200 mm and a minimum peak lower that 0.010 mm.

**Figure 8.**
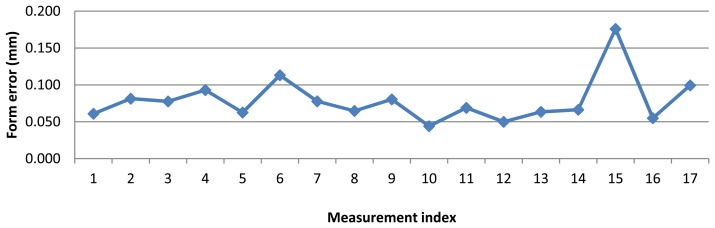
Form error for test measurements with AACMM.

Form error of the ring gauge measurement with AACMM is shown in Figure 8. Form errors vary from 0.045 to 0.175 mm. Also, a considerably measurement variability was noted.

**Figure 9.**
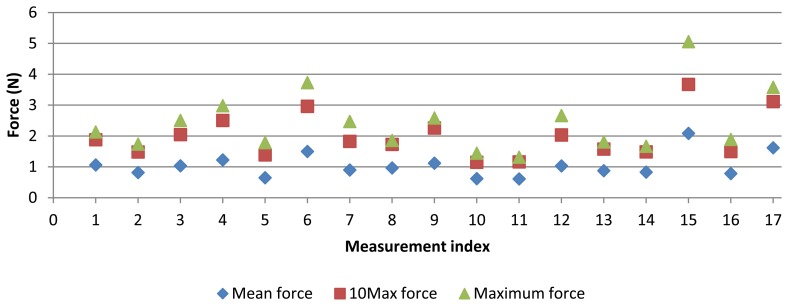
Contact force values for test measurements.

Additionally to measurement results, contact force for each point was also collected (Figure 9). For each measurement, significant values were calculated in order to obtain only one value to be compared to diameter or form error of the ring gauge. Such values were: mean force, maximum force and mean of 10 maximum contact force values (10Max). Mean value represents the force level applied during a measurement. Maximum force would probably cause the largest error. The 10Max value represents a force value of the most significant force values. Figure 9 shows these force values for each measurement. Force values also show the variability of the AACMM parameters. Maximum force reaches 5 N, but common values for maximum force are around 2.5 N. As regards mean force, it reaches up to 2 N and common values are around 1 N. 10Max force provides force values between maximum and mean force values.

**Figure 10.**
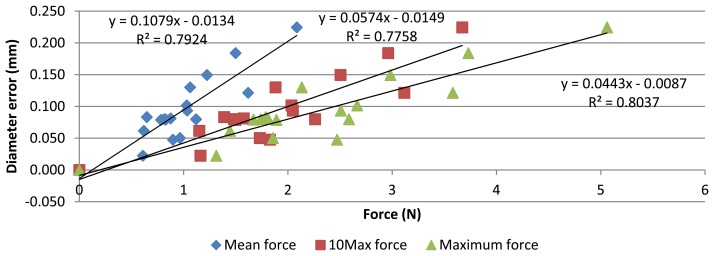
Relationship between diameter errors and contact force values.

Measurement and contact force data let to study the influence of contact force on AACMM reliability. Relationship between the force applied during measurements and diameter error can be observed in Figure 10. Determination coefficient (R^2^) for correlation study indicates that about the 78% of diameter error variation is explained by variation of the contact force, so a strong linear correlation was found. In addition, Pearson coefficients of 0.89, 0.88 and 0.90 also show a significant linear relationship with mean, 10Max and Maximum force, respectively. As aforementioned, an extra point (0 N–0 mm diameter error) was added as the reference values were taken from the CMM measurement.

**Figure 11.**
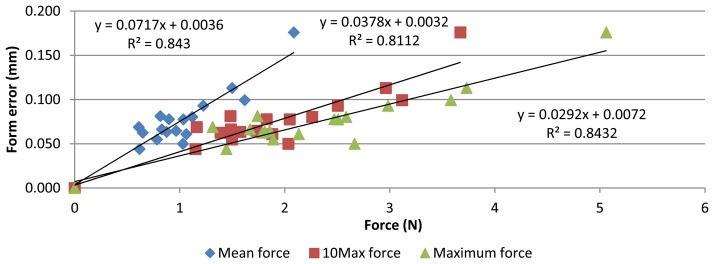
Relationship between form error and contact force values.

The same approach was used to analyse form error. Its relationship with contact force is shown in Figure 11. Again, a strong linear correlation is observed, with Pearson coefficients of 0.92, 0.90 and 0.92. Contact force variation explains about the 84% of form error variation. As before, an extra point (0 N–0 mm) was added.

Although the obtained data show that contact force is not the only factor that causes error in AACMM measurements, these results indicate their strong influence. This study proves that force on probes is one of the most important error sources. It can be explained since probe deflection is not corrected by calibration and contact force causes important deflection errors.

### 4.2. Probe Deflection Model Results

**Figure 12.**
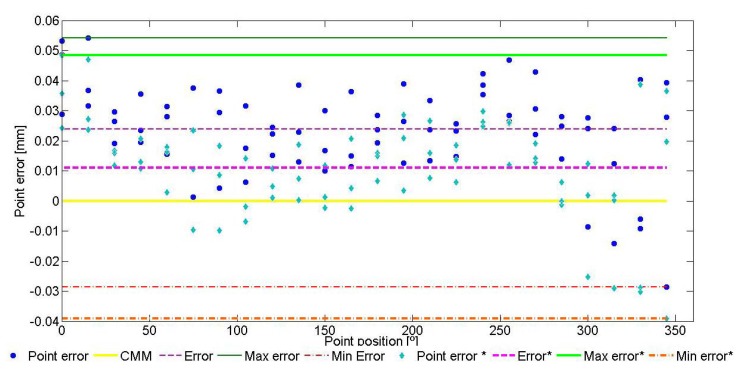
Example for contact point errors corrected with probe deflection model.

With the results of test measurements, the probe deflection model was implemented. Now, contact point coordinates are corrected with the error value caused by contact force at each point (Figure 12).

**Figure 13.**
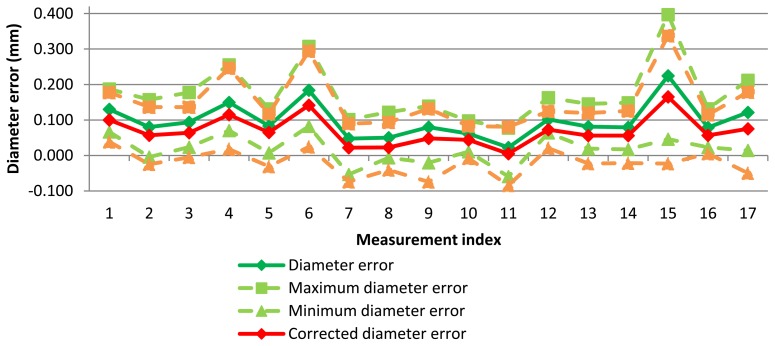
Initial and corrected diameter error for test measurements with AACMM.

In this case, each point has a lower error and, therefore, diameter error, maximum and minimum diameter errors are reduced. These diameters and form errors are calculated in the same way that measurement software does it. The probe deflection model was applied to AACMM measurements and improvement was checked (Figure 13).

The complete values for diameter error using the different probe deflection parameters are shown in Table 2. As expected, ‘*maximum force model*’ has achieved the largest reduction but in some cases this model has overcorrected the diameter error, so a lower diameter than the diameter reference was obtained. Similarly, the *‘10Max force model’* produces a significant diameter error correction but in some cases diameter has been slightly overcorrected (a few micrometers), with improvements above 100%. The models with the mean force (*mean force model*) and force of each point (*point force level*) give very similar results and a reduced diameter correction.

**Figure 14.**
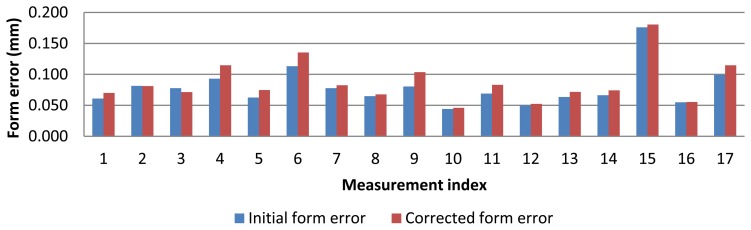
Initial and corrected form error using contact point local force.

As regard to form error, the probe deflection model that corrects each contact point with its own contact force produces a slight increase in form error (Figure 14). For the rest of models (mean, 10 Max and maximum force), form error remains the same since each point has been corrected equally, so distance between maximum and minimum radius remains unmodified.

### 4.3. Measurement Test Uncertainty Analysis

In order to validate this methodology, an uncertainty evaluation (*U_test_*), for measurements with and without probe deflection models, was carried out. This uncertainty evaluation followed GUM [33] and EA-4/02 [34] recommendations [Equation (10) ]. Expanded uncertainty is obtained as the combination of uncertainty terms that contribute to measurement uncertainty as well as the coverage factor for a level of confidence of 95%:

(10) $$U_{\text{test}}=k\cdot \sqrt{u_{\text{ring}}^{2}+u_{r}^{2}+u_{t}^{2}+u_{\text{res}}^{2}+u_{\text{cor}}^{2}}$$

Two types of uncertainty could be consider, one for dimensional error and one for form error. Standard uncertainty of the ring gauge diameter (u_ring(diameter)_) is obtained from its calibration certificate for diameter uncertainty [Equation (11) ]. The lack of information about the form error uncertainty is solved by repeated measurement, 10 repetitions, of form error of the ring gauge with the CMM. This way, the standard uncertainty of the ring gauge is the combination of the standard deviation of the mean of the form error results and the uncertainty due to the coefficient of thermal expansion of the ring gauge material (1 × 10^−6^ °C^−1^) and temperature variation (0.2 °C) [Equation (12) ]:

(11) $$u_{\text{ring}(\text{diameter})}=\frac{U_{\text{ring}(\text{diameter})}}{k}$$

(12) $$u_{\text{ring}(\text{form error})}=\sqrt{\frac{s^{2}(\text{x})}{n}+{\left(L\Delta Tu(\alpha )\right)}^{2}}$$

Standard uncertainty of repeatability (u_r_) of measurement is calculated as standard deviation of the mean value of 17 repetitions for diameter and error form measurement with the AACMM [Equation (13) ]:

(13) $$u_{\text{r}}=\sqrt{\frac{\text{s}(\text{x})}{\text{n}}}=\sqrt{\frac{\frac{1}{n-1}\sum_{i=1}^{n}{\left(x_{i}-\bar{x}\right)}^{2}}{n}}$$

Standard uncertainty of test temperature (u_t_) is caused by uncertainty of the coefficient of thermal expansion of the ring gauge and temperature variation during AACMM measurements (0.5 °C), [Equation (14) ]. L refers to the evaluated distance (diameter or form error):

(14) $$u_{\text{t}}=L\;\Delta T\;u(\alpha )$$

Standard uncertainty of resolution of the measurement system is also considered. Resolution of measurement software is 0.0001 mm and it is associated to a rectangular distribution [Equation (15) ]:

(15) $${\text{u}}_{\text{res}}=\frac{\text{resolution}}{\sqrt{12}}$$

Standard uncertainty of the correction model is calculated with the uncertainty of contact force sensor and FEM analysis [Equation (16) ]. If the probe deflection model is not applied this term has no effect. Due to ring geometry, standard uncertainty of the correction is considered as two times the uncertainty for probe deflection:

(16) $$u_{\text{cor}}=2\cdot 0.0142\cdot u_{\text{sensor}}$$

Uncertainty budget for AACMM measurements without and with probe deflection correction are shown in Table 3. Uncertainty results show that the force contact sensor and correction model are suitable for AACMM.

## 5. Conclusions

A contact force sensor for AACMM measurements has been developed. This sensor allows the evaluation of contact force on AACMM performance. Furthermore, a test methodology was designed in order to study the relationship between contact force parameters and diameter and form error of a ring gauge. A strong relationship (determination coefficient higher than 80%) has been found and furthermore, the contribution of contact force to AACMMM performance can reach up to 0.200 and 0.150 mm for diameter and form error, respectively, according to our contact force regression study. As a result, contact force has been proved to be one of the main factors that affects AACMM reliability.

In order to reduce diameter error, a probe deflection model based on the geometry of the ring gauge and contact force parameters has been proposed and implemented. As a result, the probe deflection model has given significantly lower diameter errors for the ring gauge test. Diameter error values have reached about 30%, 30%, 60% and 55% improvement, respectively, for each probe deflection model, that is, point, *mean force*, *10Max force* and *max force* model, respectively, with regard to initial diameter errors attained by AACMM measurements. For purposes of validation of the test and probe deflection model an uncertainty evaluation was carried out. Results of this evaluation indicate that the sensor system and the correction models are suitable, since variability of the measured data has decreased.

With this survey not only have the errors induced by probe deformation been evaluated, but also a methodology has been developed to increase the reliability. In any case, a commercial version of this sensor could evaluate the operator performance during the measuring by establishing a methodology to provide “force warnings” or to correct measurements. These features should be incorporated in the next generation of AACMMs.

## Figures and Tables

**Table 1.** Uncertainty budget for contact force sensor system.

**Term**	**Type**	**Distribution**	**d.o.f.**	**Sensibility Coef.**	**Uncertainty Contribution**	

					**Axial [N]**	**Bending [N]**
u_stw_	B	Rectangular	∞	1	0.0040	0.0040
u_cre_	B	Rectangular	∞	1	0.0001	0.0001
u_Eo_	B	Rectangular	∞	1	0.0081	0.0081
u_res_	B	Rectangular	∞	1	0.0003	0.0003
u_hys_	B	Rectangular	∞	1	0.0380	0.0550
u_rep_	A	Normal	9	1	0.0250	0.0460
u_tem_	B	Rectangular	∞	LΔT	0.0000	0.0000

**Combined Standard Uncertainty**					0.0464	0.00723
**Effective Degrees of Freedom**					29	29
**Coverage Factor k**					2,1	2,1
**Expanded Uncertainty U_sensor(95%)_**					**0.0974**	**0.1518**

**Table 2.** Improvement of diameter error for tested deflection models.

**Measurement**	**Diameter Error (mm) (Improvement (%))**				

	**Initial**	**Point Force**	***Mean Force***	***Max. Force***	***10Max Force***
**1**	0.130	0.100 (23)	0.100 (23)	0.070 (46)	0.077 (41)
**2**	0.080	0.057 (29)	0.057 (29)	0.031 (61)	0.038 (53)
**3**	0.093	0.064 (31)	0.064 (31)	0.022 (76)	0.035 (62)
**4**	0.150	0.110 (27)	0.115 (23)	0.065 (57)	0.078 (48)
**5**	0.083	0.065 (22)	0.065 (22)	0.033 (60)	0.044 (47)
**6**	0.184	0.142 (23)	0.141 (23)	0.078 (58)	0.100 (46)
**7**	0.048	0.023 (52)	0.022 (54)	−0.022 (146)	−0.004 (108)
**8**	0.050	0.023 (54)	0.023 (54)	−0.002 (104)	0.001 (98)
**9**	0.080	0.048 (40)	0.048 (40)	0.008 (91)	0.016 (80)
**10**	0.062	0.044 (29)	0.044 (29)	0.021 (66)	0.029 (53)
**11**	0.023	0.005 (78)	0.005 (78)	−0.015 (165)	−0.010 (143)
**12**	0.102	0.073 (28)	0.072 (29)	0.026 (75)	0.044 (57)
**13**	0.081	0.056 (31)	0.056 (31)	0.030 (63)	0.036 (56)
**14**	0.079	0.056 (29)	0.056 (29)	0.032 (59)	0.037 (53)
**15**	0.225	0.165 (27)	0.165 (27)	0.081 (64)	0.120 (47)
**16**	0.079	0.057 (28)	0.057 (28)	0.025 (68)	0.036 (54)
**17**	0.121	0.075 (38)	0.076 (37)	0.020 (83)	0.033 (73)
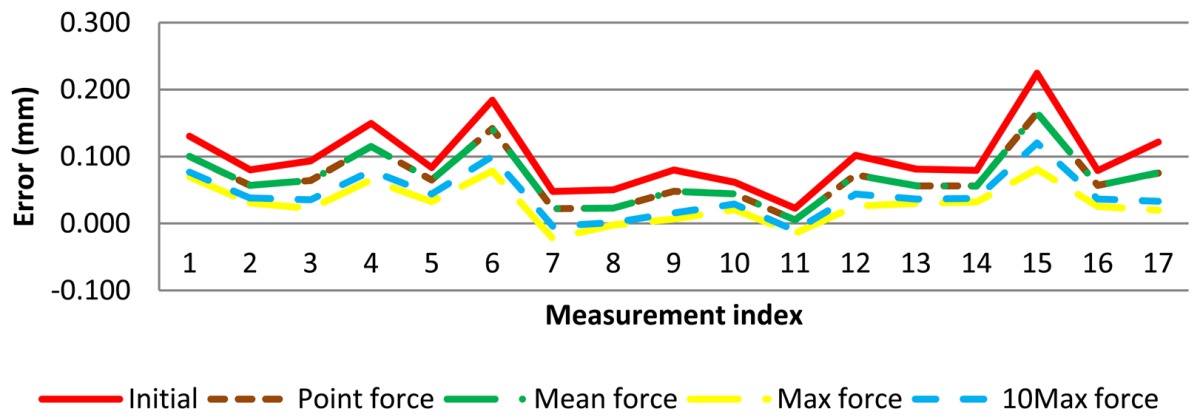					

**Table 3.** Uncertainty budget with and without probe deflection compensation model.

**Term**	**Type**	**Distribution**	**d.o.f.**	**Sensibility Coef.**	**Uncertainty without Model**		**Uncertainty with Model**	

					**Diameter (mm)**	**Form error (mm)**	**Diameter (mm)**	**Form error (mm)**
*u_ring_*	B	Rectangular	∞	1	0.00105	0.00230	0.00105	0.00230
*u_r_*	A	Normal	16	1	0.01233	0.00746	0.01012	0.00821
*u_t_*	B	Rectangular	∞	LΔT	0.00009	1.3 × 10^−9^	0.00009	1.3 × 10^−9^
*u_res_*	B	Rectangular	∞	1	0.00003	0.00003	0.00003	0.00003
*u_cor_*	B	Rectangular	∞	2·0.0142	-	-	0.00132	0.00132

Combined uncertainty					0.01237	0.00781	0.01026	0.00863
Effective degree of freedom					16	16	16	16
Coverage factor k					2.15	2.15	2.15	2.15
Expanded uncertainty U_test (95%)_					**0.02660**	**0.01679**	**0.02206**	**0.01855**
